# An unusual case of high hyperdiploid childhood ALL with cryptic *BCR*/*ABL1* rearrangement

**DOI:** 10.1186/s13039-014-0072-9

**Published:** 2014-10-24

**Authors:** Libuse Lizcova, Zuzana Zemanova, Halka Lhotska, Jan Zuna, Lenka Hovorkova, Ester Mejstrikova, Eva Malinova, Jana Rabasova, Ivan Raska, Lucie Sramkova, Jan Stary, Kyra Michalova

**Affiliations:** Center of Oncocytogenetics, Institute of Medical Biochemistry and Laboratory Diagnostics, General University Hospital in Prague and First Faculty of Medicine, Charles University in Prague, Prague, Czech Republic; CLIP-Childhood Leukaemia Investigation Prague, Department of Paediatric Haematology and Oncology, Second Faculty of Medicine, Charles University, Prague and University Hospital Motol, Prague, Czech Republic; Department of Medical Genetics, Faculty Hospital Hradec Kralove, Hradec Kralove, Czech Republic; Institute of Cellular Biology and Pathology, First Faculty of Medicine, Charles University in Prague, Prague, Czech Republic; Department of Paediatric Haematology and Oncology, Second Faculty of Medicine, Charles University, Prague and University Hospital Motol, Prague, Czech Republic

**Keywords:** High-hyperdiploid childhood ALL, Ph-negative childhood ALL, *BCR*/*ABL1* fusion, FISH, CGH–SNP array

## Abstract

**Background:**

Both high hyperdiploidy (HeH) and the translocation t(9;22)(q34;q11) are recurrent abnormalities in childhood B-cell acute lymphoblastic leukemia (ALL) and both are used in current classification to define different genetic and prognostic subtypes of the disease. The coexistence of these two primary genetic aberrations within the same clone is very rare in children with ALL. Here we report a new case of a 17-year-old girl with newly diagnosed ALL and uncommon cytogenetic and clinical finding combining high hyperdiploidy and a cryptic *BCR*/*ABL1* fusion and an inherited Charcot-Marie-Tooth neuropathy detected during the induction treatment.

**Results:**

High hyperdiploid karyotype 51,XX,+X,+4,+14,+17,+21 without apparent structural aberrations was detected by conventional cytogenetic analysis and multicolor FISH. A cryptic *BCR*/*ABL1* fusion, which was caused by the insertion of part of the *ABL1* gene into the 22q11 region, was proved in HeH clone by FISH, RT-PCR and CGH-SNP array. In addition, an abnormal FISH pattern previously described as the deletion of the 3′*BCR* region in some *BCR*/*ABL1* positive cases was not proved in our patient.

**Conclusion:**

A novel case of extremely rare childhood ALL, characterized by HeH and a cryptic *BCR*/*ABL1* fusion, is presented and to the best of our knowledge described for the first time. The insertion of *ABL1* into the *BCR* region in malignant cells is supposed. Clearly, further studies are needed to determine the genetic consequences and prognostic implications of these unusual cases.

## Background

Pediatric B-cell precursor acute lymphoblastic leukemia (BCP-ALL) is a heterogeneous disease on both the cytogenetic and genetic levels [1]. A number of acquired chromosomal aberrations have been identified in the bone marrow cells of children with ALL, which provide diagnostic and prognostic information that directly affects patient management [2].

The Philadelphia (Ph) chromosome, i.e., translocation t(9;22)(q34;q11), giving rise to the *BCR*/*ABL1* fusion gene, is a rare finding in children with ALL, accounting for ~3% of cases, and is traditionally associated with a poor outcome [3]. However, implementation of tyrosine kinase inhibitors to standard therapy greatly improved the survival of children with Ph + ALL [4]. In a small number of ALL patients, the Ph chromosome is not detected by conventional cytogenetics, but the *BCR*/*ABL1* fusion is present [5]. In these cases, the fusion either arises as a cryptic rearrangement or is masked within a complex karyotype and can only be detected by molecular cytogenetic and/or molecular genetic methods. On the contrary, high hyperdiploidy (HeH), defined as the presence of 51–67 chromosomes in the karyotype, is the most frequent cytogenetic finding in childhood ALL, occurring in 25%–30% of cases. It is characterized by a nonrandom gain of specific chromosomes and a clinically favorable prognosis [6,7].

Although HeH is a common finding in childhood ALL, there are rare instances of high-hyperdiploid patients carrying other ALL-specific translocations, i.e., t(9;22)(q34;q11), t(12;21)(p13;q22), t(1;19)(q23;p13), and *MLL* rearrangements. These cases comprise 1%–4% of the patients with high-hyperdiploid ALL [8]. We present a patient with newly diagnosed ALL and a rare cytogenetic finding, combining HeH and an unusual *BCR*/*ABL1* fusion caused by the insertion of part of the *ABL1* gene into the 22q11 region. Moreover, the patient was diagnosed with an inherited neuropathy Charcot-Marie-Tooth syndrome during the antileukemic treatment.

## Case presentation

A 17-year-old girl was diagnosed with common ALL (cALL) in January 2013 after experiencing one month of fatigue, bone pain at rest, and lymphocytosis in the peripheral blood (lymphocytes 86%). A complete blood count showed 11.3 × 10^9^/L WBC, 86 g/L hemoglobin, and a platelet count of 42 × 10^9^/L. Her bone marrow was infiltrated by lymphoblasts (96.8%) with an L1 morphology. Blasts had hyperdiploid DNA content (DNA index 1.089). Immunophenotypically blasts corresponded to cALL with aberrant expression of CD66c, high CD34 positivity and low expression of CD38 antigen. Immunophenotype was typical for BCR/ABL1 positivity [9]. BCR/ABL fusion gene was confirmed with multiplex RT-PCR.

The patient has been treated according to the EsPhALL 2010 protocol for BCR/ABL-positive ALL with a combination of chemotherapy and imatinib (Glivec). During induction, the patient developed very severe peripheral neurotoxicity with quadriparesis and paralytic ileus, which required major surgery with stoma. This critical clinical condition resulted in interruption of the chemotherapy treatment for two weeks. Surprisingly, the cause of this unexpected complication was inherited neuropathy Charcot-Marie-Tooth syndrome (CMT1A subtype), with a proven PMP22 mutation. Subsequently it was clear that the neurotoxicity was an abnormal reaction to Vincristine therapy and therefore further vinca alkaloid medication was contraindicated. At present, the patient is in continuous complete remission (18 months from the diagnosis) on maintenance chemotherapy with imatinib, she is after submerging of her stoma and her neurological status is improving.

For the cytogenetic analyses, bone marrow cells were cultured for 24 hours without stimulation, and chromosomal preparations were made using standard techniques. In total, 25 metaphases were analyzed and the karyotypes were described according to An International System for Human Cytogenetic Nomenclature (ISCN 2013) [10].

To detect the *BCR*/*ABL1* fusion gene, fluorescence in situ hybridization (FISH) was performed with commercially available locus-specific probes (Vysis LSI BCR/ABL Dual Color Dual Fusion Translocation Probe and Vysis LSI BCR/ABL ES Dual Color Translocation Probe; Abbott Molecular, Des Plaines, IL, USA). All available metaphases and 200 interphase nuclei were analyzed.

Other chromosomal aberrations were analyzed with multicolor FISH (mFISH) and comparative genomic hybridization based array (aCGH) using the 24*X*Cyte color kit (MetaSystems, Altlussheim, Germany) and an oligonucleotide CGH–single-nucleotide polymorphism (SNP) array (BlueGnome, Cambridge, UK) respectively. Array results were confirmed by FISH with the appropriate bacterial artificial chromosome (BAC) probes (BlueGnome). All techniques were done according to manufacturer’s recommendations.

The fusion transcript *BCR*/*ABL1* was detected with reverse transcription–polymerase chain reaction (RT–PCR), which was performed according to Europe Against Cancer program protocol [11]. The amount of cDNA was normalized to the expression of housekeeping gene GUS as described previously [12].

**Figure 1:**
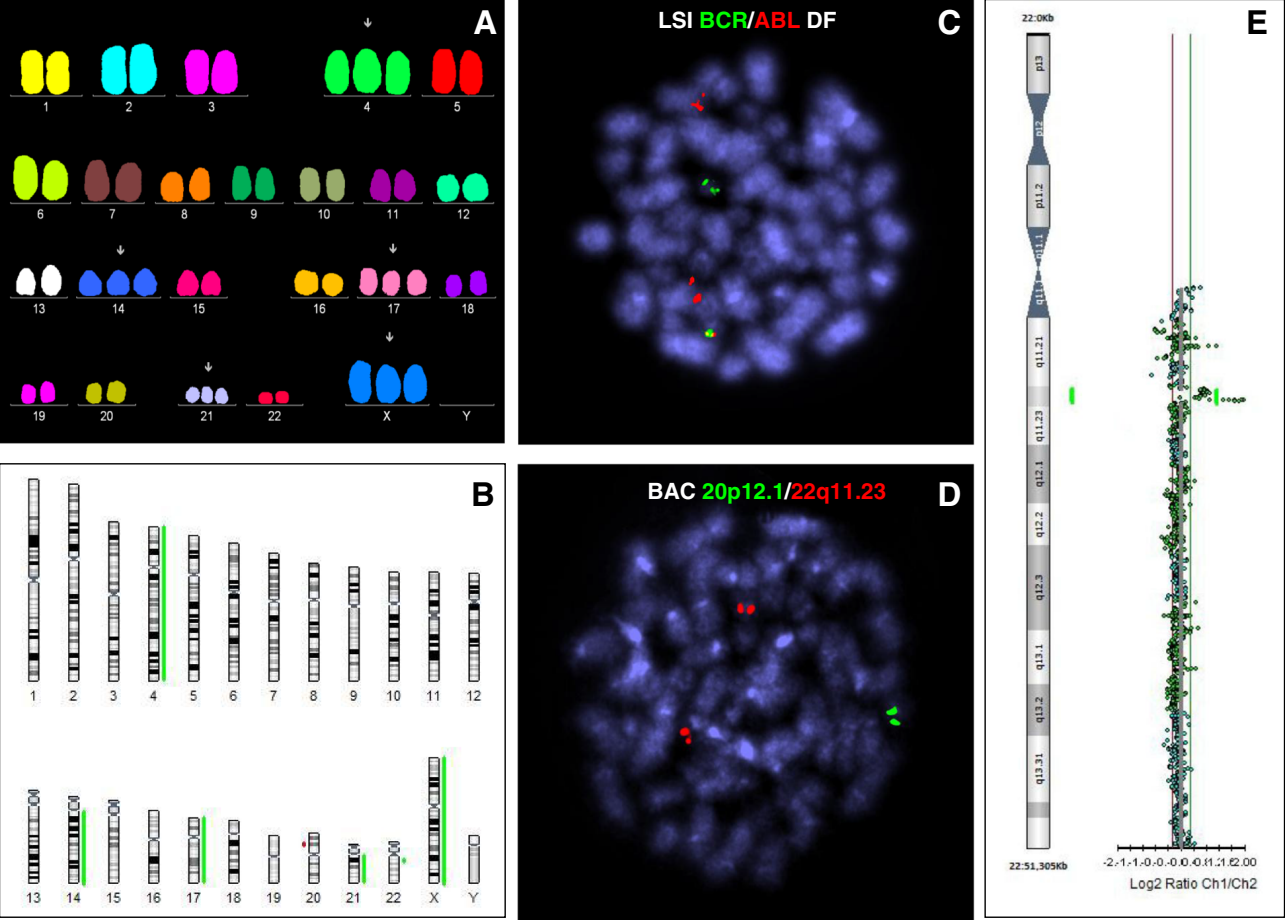
**Results of molecular cytogenetic analysis.** Multicolor FISH **(A)** and array CGH **(B)** showing trisomies of chromosomes X, 4, 14, 17 and 21 **(A, B)** and submicroscopic aberrations: deletion of chromosome 20 and amplification of chromosome 22 **(B)**. BCR/ABL1 positive metaphase hybridized with BCR/ABL dual fusion (DF) probe demonstrating apparent loss of green 3′BCR signal, i.e. 1F2O1G FISH pattern (caused by the insertion of the *ABL1* gene into the *BCR* region, thus entire BCR signal remained on chromosome 22) **(C)**. FISH with BAC probes RP11-80O7 (orange) and RP11-400P21 (green) with evidence of normal finding of 22q11.23 region matching to 3′BCR locus (two orange signals) and deletion of 20p12.1 (one green signal) **(D)**. Detailed view of array CGH result of chromosome 22 showing amplification of the 22q11.22 region and normal 22q11.23 region pattern **(E)**.

An m-*BCR*/*ABL1* fusion gene was detected with multiplex RT–PCR. Moreover, we found also the genomic breakpoint between the *BCR* and *ABL1* genes (*BCR* intron 1/*ABL1* intron 1). The karyotype was described as follows: 51,XX,+X,+4,+14,+17,+21[22]/46,XX[3]. However, FISH with the BCR/ABL Extra Signal and BCR/ABL Dual Fusion Probes detected the *BCR*/*ABL1* fusion with an abnormal FISH signal pattern (1F2O1G), consistent with a breakpoint within m-*BCR* and the loss of the 3′*BCR* signal from der(9) in ten out of eleven metaphases and ~70% of the interphase nuclei (Figure 1). A detailed FISH signal analysis of the abnormal metaphases revealed the fusion signal to be located on apparently normal chromosome 22 and the *ABL1* signal in the 9q34 region of two copies of chromosome 9. The expected deletion of the 3′*BCR* region (from the FISH results) was not detected by array CGH and BAC probes and only other chromosomal abnormalities were found, including trisomies of chromosomes X, 4, 14, 17, and 21, cryptic deletion of the short arm of chromosome 20 [del (20) (p12.1p12.1)] and submicroscopic amplification of the 22q11.22 region (Figure 1).

Based on the cytogenetic and genetic results, it is clear that the *BCR*/*ABL1* fusion in our patient arose from the submicroscopic insertion of part of the *ABL1* gene into chromosome 22. It is based on the facts that: (1) no classical or variant Ph translocation was detected by conventional cytogenetic/mFISH; (2) loss of the 3′*BCR* region, although expected from interphase FISH signal pattern, was not confirmed by array CGH or by FISH with BAC probes for this region, i.e. entire BCR signal remained on chromosome 22 and was not translocated on chromosome 9; and (3) only the *BCR*/*ABL1* fusion was proved at the RNA as well as DNA level, while the reciprocal *ABL1*/*BCR* fusion was not found by any of these approaches.

Our results show that only parallel cytogenetic, molecular cytogenetic, and molecular genetic analyses can provide detailed information about cryptic and prognostically significant aberrations that could not have been achieved with any of these techniques used independently. The metaphase FISH results were important for the correct interpretation of the interphase FISH findings, because only this analysis could identify the location of the *BCR*/*ABL1* fusion on der(22). The current inclusion of array techniques into routine practice could lead us to reevaluate the real occurrence of deletions of either the 3′*BCR* or 5′*ABL1* region in *BCR*/*ABL1*-positive cases (especially with cryptic insertions) previously described by FISH analyses.

Several patients with CML, or less frequently with ALL, have been reported with cryptic insertions of part of the *BCR* region into *ABL1* at 9q34 or rarely of *ABL1* into *BCR* at 22q11 [13,14]. However, to our knowledge, this is the first published case of such an aberration in childhood high-hyperdiploid ALL with inherited Charcot-Marie-Tooth neuropathy. In general, ALL-specific rearrangements are extremely rare in patients with HeH and it is difficult to identify the primary abnormality. In most cases (as in our patient), both aberrations were presented simultaneously in abnormal cells. However, few cases of HeH Ph-positive ALL have been published in the literature [15,16], and these patients displayed not only a Ph-positive HeH clone, but also cells with 46 chromosomes and t(9;22)(q34;q11) as the sole abnormality, suggesting that the Ph chromosome was the primary aberration.

There is no difference in the gain of specific chromosomes (namely X, 4, 6, 8, 10, 14, 17, 18 a 21) between patients with high-hyperdiploid ALL and commonly encountered ALL aberrations, and those with high-hyperdiploid ALL. The only exception is the trisomy of chromosome 2, which was previously described as a quite frequent chromosome gain in patients with translocation t(9;22)(q34;q11) [6,16]. However, trisomy of chromosome 2 was not found in our patient, nor was revealed as a frequent trisomy in six other currently described cases with Ph positive HeH ALL [8].

From a clinical perspective, patients with HeH and well-known translocations are considered to represent a biologically distinct subgroup, which may have independent prognostic implications [17]. From previously reported cases, it seems that the prognostic impact of the translocation could override the beneficial effect of HeH [17,18]. However, brief follow-up period does not allow to assess any conclusion about the impact of this finding on prognosis in our patient nor to predict the effect of the inherited neuropathy.

## Conclusion

In conclusion, a novel case of extremely rare childhood ALL, characterized by HeH and a cryptic *BCR*/*ABL1* fusion, is presented and described for the first time and the insertion of *ABL1* into the *BCR* region in malignant cells is supposed. Although several cases of childhood ALL with HeH and general ALL-specific aberrations have been published, the exact pathogenetic mechanisms of the disease in these patients have not been clarified. Clearly, further studies of these uncommon cases are necessary to determine the genetic consequences and real prognostic implications of these phenomena.

## Consent

Written informed consent was obtained from the patient’s parents for publication of this Case report and any accompanying images. A copy of the written consent is available for review by the Editor-in-Chief of this journal.

## Abbreviations

BCP-ALL: B-cell precursor acute lymphoblastic leukemia
CML: Chronic myeloid leukemia
HeH: High hyperdiploidy
FISH: Fluorescence in situ hybridization
mFISH: Multicolor FISH
RT-PCR: Reverse transcription-polymerase chain reaction
CGH-SNP array: Comparative genomic hybridization-single nucleotide polymorphism array
BAC probes: Bacterial artificial chromosome probes
